# Smoking cessation interventions in South Asian Region: a systematic scoping review

**DOI:** 10.1186/s12889-022-13443-y

**Published:** 2022-06-01

**Authors:** Sajid Iqbal, Rubina Barolia, Pammla Petrucka, Laila Ladak, Rameesha Rehmani, Abdul Kabir

**Affiliations:** 1 The Aga Khan University, Karachi, Pakistan; 2 Aga Khan University School of Nursing and Midwifery, Karachi, Pakistan; 3 College of Nursing University of Saskatchewan, Saskatoon, Saskatchewan Canada; 4 University of Toronto, Toronto, Canada

**Keywords:** Tobacco, Cigarette, Quit*, Cessation, Strategies, Interventions, Measures, South Asia*

## Abstract

**Background:**

Cigarette smoking is one of the most preventable causes of morbidities and mortalities. Since 2005, the World Health Organization Framework Convention for Tobacco Control (WHO-FCTC) provides an efficient strategic plan for tobacco control across the world. Many countries in the world have successfully reduced the prevalence of cigarette smoking. However, in developing countries, the prevalence of cigarette smoking is mounting which signifies a need of prompt attention. This scoping review aims to explore the extent and nature of Smoking Cessation (SmC) interventions and associated factors in South Asian Region (SAR) by systematically reviewing available recently published and unpublished literature.

**Methods:**

The Joanna Briggs Institute (JBI) framework frames the conduct of this scoping review.

PubMed, EBSCO CINAHL Complete, Cochrane Library, ProQuest Dissertation and Theses, and local websites as well as other sources of grey literature were searched for relevant literature. In total, 573 literature sources were screened. Following the Preferred Reporting Items for Systematic Reviews and Meta-Analyses (PRISMA) flow diagram, finally, 48 data sources were included for data extraction and analysis.

We analyzed the extracted SmC interventions through the FCTC. Factors that affect smoking cessation interventions will be extracted through manual content analysis.

**Results:**

Regarding FCTC recommended smoking cessation strategies (articles), most of the articles were either neglected or addressed in a discordant way by various anti-smoking groups in SAR. Key barriers that hamper the effectiveness of smoking cessation interventions included lack of awareness, poor implementation of anti-smoking laws, and socio-cultural acceptance of tobacco use. Conversely, increased levels of awareness, through different mediums, related to smoking harms and benefits of quitting, effective implementation of anti-smoking laws, smoking cessation trained healthcare professionals, support systems, and reluctance in the community to cigarette smoking were identified as facilitators to smoking cessation interventions.

**Conclusion:**

The ignored or uncoordinated FCTC’s directions on smoking cessation strategies have resulted in continued increasing prevalence of cigarette smoking in developing countries, especially SAR. The findings of this review highlight the need for refocusing the smoking cessation strategies in SAR.

**Strengths:**

The review was conducted by a team of expert comprising information specialists, and senior professors bringing rich experience in systematic and scoping reviews. Every effort was made to include all available literature sources addressing cigarette SmC and associated factors in SAR. The review findings signal the need and direction for more SmC efforts in SAR which may contribute to development of effective policies and guidelines for the control of smoking prevalence.

**Limitations:**

Despite efforts, potentially relevant records may have been missed due to unpublished or inaccessible articles, unintended selection bias, or those published in local languages, etc. Moreover, the exclusion of literature on under 18 participants and mentally ill smokers may limit the generalizability of findings.

**Supplementary Information:**

The online version contains supplementary material available at 10.1186/s12889-022-13443-y.

## Background

Tobacco-associated diseases are the first human-created global epidemic [1]. Tobacco use has resulted in 100 million lives lost in the twentieth century, with estimates of 1 billion more in the twenty-first century if the existing patterns of tobacco use remain unchanged [2]. More than 80% of the world’s tobacco users reside in developing countries like south Asian countries [3]. Tobacco use causes the death of 1.2 million people in SAR [4]. The updated data on prevalence of smoking in SAR were not found. However, according to data produced in 2009 and 2014, the burden of tobacco smoking among men was found 43% in Bangladesh, 42% in Maldives, 34.6% in India, 33% in Pakistan, 32% in Nepal, and 29% in Sri Lanka [4, 5]. Due to socio-cultural factors, the cigarette smoking prevalence is higher amongst men as compare to women in SAR except Nepal. In Nepal almost 26% amongst female smoke tobacco [4].

Cigarette smoking leads to detrimental health issues including cancer, cardiovascular, and pulmonary diseases [1] along with harmful effects of smoking on non-smokers [6]. Tobacco use contributes to poverty by usurping household expenses from basic needs like food, education, and shelter. Additionally, tobacco-associated diseases and deaths create economic damage due to healthcare costs and loss of human capital [3]. Individuals experiencing tobacco-related chronic health issues, mainly cancer, cardiovascular, and pulmonary diseases, are more motivated for smoking cessation (SmC) with an odds ratio ranging from 1.22 (95% CI 0.91–1.63) to 13.28 (95% CI 8.45–20.88) [7, 8].

Growing prevalence and consequences of smoking warrant attention to SmC. The World Health Assembly adopted the World Health Organization’s Framework Convention for Tobacco Control (WHO-FCTC) in 2003 with implementation in 2005. In 2018, FCTC was identified as an extensively adopted tobacco control framework amongst the United Nations signatories. The FCTC assists member countries in combating the tobacco epidemic through a comprehensive collection of evidence-based measures across a number of domains (e.g. reducing tobacco demand/supply) [9–11]. Through implementation of the main five [of 22] FCTC propositions (referred to as *articles*), one study found a significant (*p*-0.001) mean difference in smoking prevalence between 2005 and 2015 [12]. Hence, FCTC is a reliable framework to examine the level of initiatives taken by a country to reduce smoking prevalence. Several member countries have devised policies, laws, and guidelines to implement the FCTC articles and progress towards tobacco reduction targets. However, many low- and middle-income countries struggle with the effective and practical adaptation of FCTC, and are unlikely to achieve the target set by WHO that is 30% reduction in tobacco use by 2025 [11, 13].

### Rationale

Ascertaining the effectiveness of similar SmC interventions in developed and developing countries has been challenging. Smoking cessation interventions, like anti-tobacco campaigns via mass media; increased cost of tobacco; widespread smoke-free regulations; accessible SmC support programs; and smoking health hazards warnings in films, have been effective in developed countries. The United States of America (USA) reduced smoking prevalence from 20.9 to 15.5% between 2005 and 2016; but the same trend has not been achieved in developing countries [8, 14, 15]. Countries in the South Asian Region (SAR) have utilized different SmC strategies; some are based on FCTC or MPOWER; others are unique to Asian cultural and societal norms.

Our search did not find any review conducted on a range of SmC interventions or facilitators and barriers to SmC in SAR. Reviews conducted in a specific country of SAR (e.g. India) included either trialed interventions or non-systematic search strategies that limited the inclusion of a broader range of available literature [16, 17].

Internationally, systematic reviews [18–22] and scoping reviews [23–25] have been limited to either inclusion of specific types of studies or to specific SmC interventions, often reflecting on technology based interventions, efficacy/effectiveness of interventions, or target populations for interventions. Clearly there is a lack of a unified range of SmC interventions and associated factors in literature from SAR.

### Objectives

This scoping review explores the extent and nature of interventions for SmC in SAR by systematically reviewing available recently published and unpublished literature. It will seek factors that hinder or facilitate SmC interventions in SAR.

## Methodology

This scoping review was registered with the Joanna Briggs Institute (JBI) register for systematic reviews page 10, dated 28th January 2020. Furthermore, the protocol for this review was published in the British Medical Journal Open (BMJ-Open) on January 2021 [26]. To ensure the inclusion of required components, we used Preferred Reporting Items for Systematic reviews and Meta-Analyses extension for Scoping Reviews (PRISMA-ScR) Checklist (please refer to Table 1).

**Table 1:** Preferred Reporting Items for Systematic reviews and Meta-Analyses extension for Scoping Reviews (PRISMA-ScR) checklist

Section	Item	PRISMA-ScR checklist item	Reported on page #
**Title**			
Title	1	Identify the report as a scoping review.	1
**Abstract**			
Structured summary	2	Provide a structured summary that includes (as applicable): background, objectives, eligibility criteria, sources of evidence, charting methods, results, and conclusions that relate to the review questions and objectives.	2–3
**Introduction**			
Rationale	3	Describe the rationale for the review in the context of what is already known. Explain why the review questions/objectives lend themselves to a scoping review approach.	5–6
Objectives	4	Provide an explicit statement of the questions and objectives being addressed with reference to their key elements (e.g., population or participants, concepts, and context) or other relevant key elements used to conceptualize the review questions and/or objectives.	6
**Methods**			
Protocol and registration	5	Indicate whether a review protocol exists; state if and where it can be accessed (e.g., a Web address); and if available, provide registration information, including the registration number.	6
Eligibility criteria	6	Specify characteristics of the sources of evidence used as eligibility criteria (e.g., years considered, language, and publication status), and provide a rationale.	6
Information sources^a^	7	Describe all information sources in the search (e.g., databases with dates of coverage and contact with authors to identify additional sources), as well as the date the most recent search was executed.	6–7
Search	8	Present the full electronic search strategy for at least 1 database, including any limits used, such that it could be repeated.	6–7
Selection of sources of evidence^b^	9	State the process for selecting sources of evidence (i.e., screening and eligibility) included in the scoping review.	6–7
Data charting process^c^	10	Describe the methods of charting data from the included sources of evidence (e.g., calibrated forms or forms that have been tested by the team before their use, and whether data charting was done independently or in duplicate) and any processes for obtaining and confirming data from investigators.	8
Data items	11	List and define all variables for which data were sought and any assumptions and simplifications made.	8
Critical appraisal of individual sources of evidence^d^	12	If done, provide a rationale for conducting a critical appraisal of included sources of evidence; describe the methods used and how this information was used in any data synthesis (if appropriate).	NA
Synthesis of results	13	Describe the methods of handling and summarizing the data that were charted.	8
**Results**			
Selection of sources of evidence	14	Give numbers of sources of evidence screened, assessed for eligibility, and included in the review, with reasons for exclusions at each stage, ideally using a flow diagram.	Table 1
Characteristics of sources of evidence	15	For each source of evidence, present characteristics for which data were charted and provide the citations.	8–10
Critical appraisal within sources of evidence	16	If done, present data on critical appraisal of included sources of evidence (see item 12).	NA
Results of individual sources of evidence	17	For each included source of evidence, present the relevant data that were charted that relate to the review questions and objectives.	Supp. Material I & II
Synthesis of results	18	Summarize and/or present the charting results as they relate to the review questions and objectives.	9–16
**Discussion**			
Summary of evidence	19	Summarize the main results (including an overview of concepts, themes, and types of evidence available), link to the review questions and objectives, and consider the relevance to key groups.	15–17
Limitations	20	Discuss the limitations of the scoping review process.	2
Conclusions	21	Provide a general interpretation of the results with respect to the review questions and objectives, as well as potential implications and/or next steps.	18
**Funding**			
Funding	22	Describe sources of funding for the included sources of evidence, as well as sources of funding for the scoping review. Describe the role of the funders of the scoping review.	20

*From:* Tricco AC, Lillie E, Zarin W, O’Brien KK, Colquhoun H, Levac D, et al. PRISMA Extension for Scoping Reviews (PRISMAScR): Checklist and Explanation. Ann Intern Med. 2018;169:467–473. doi: 10.7326/M18-0850

*JBI* Joanna Briggs Institute, *PRISMA-ScR* Preferred Reporting Items for Systematic reviews and Meta-Analyses extension for Scoping Reviews

^a^Where *sources of evidence* (see second footnote) are compiled from, such as bibliographic databases, social media platforms, and Web sites

^b^A more inclusive/heterogeneous term used to account for the different types of evidence or data sources (e.g., quantitative and/or qualitative research, expert opinion, and policy documents) that may be eligible in a scoping review as opposed to only studies. This is not to be confused with *information sources* (see first footnote)

^c^The frameworks by Arksey and O’Malley [6] and Levac and colleagues [7] and the JBI guidance [4, 5] refer to the process of data extraction in a scoping review as data charting

^d^The process of systematically examining research evidence to assess its validity, results, and relevance before using it to inform a decision. This term is used for items 12 and 19 instead of “risk of bias” (which is more applicable to systematic reviews of interventions) to include and acknowledge the various sources of evidence that may be used in a scoping review (e.g., quantitative and/or qualitative research, expert opinion, and policy document)

As mentioned in the published protocol, we followed the JBI nine steps underpinned by the framework of Arksey and O’Malley in conducting this scoping review [27, 28]. For a detailed description of the JBI nine steps for scoping review, refer to our published protocol [26].

### Literature search strategy and criteria for selection

We consulted an informational specialist [MK] respecting the selection of relevant databases and search terms. In December 2020, a systematic literature search was conducted across PubMed, EBSCO CINAHL Complete, Cochrane Library, and ProQuest Dissertation and Theses for the most recent 5 years of literature. The search was updated in June 2021 to include any additional publications to the databases (search strategy attached as supplementary material III). To enrich the extracted data, citation chaining was used to extract classic references from bibliographies of selected articles, and emailing authors of anti-smoking and/or anti-tobacco articles was also done. A grey literature search was conducted across the Canadian Agency for Drugs and Technologies in Health (CADHT), Open Grey, Blogs, and local as well global websites. Details of the search terms, mesh words, Boolean operators, wildcards, and search syntaxes are provided in the published protocol [26].

We included all types of literature on interventions and barriers or facilitators to SmC relevant to the adult SAR population published in the last 5 years. Also, literature in English or any other language with English translation available were included. While studies including participants from countries other than SAR were excluded.

All relevant citations were imported to EndNote™ software. Two independent reviewers [SI and AK] read the titles and abstracts of the imported citations. Subfolders were developed in EndNote™ where the articles approved for full-text read were separated from those deemed irrelevant. The final inclusion of an article was decided after a full read and mutual agreement. Uncertainties encountered were discussed with supervisors [RR and PP].

### Data extraction

The team developed separate templates in an Excel™ sheet for data extraction from empirical and non-empirical sources of literature. The empirical studies template extracted the study’s main characteristics like author/s name, year of publication, study settings, aim, design, framework or theory, population, smoking cessation interventions, barriers and/or facilitators, outcomes, limitations, and recommendations. From the non-empirical sources, the extracted characteristics included author/s, year of publication or the year of update, design /framework/ theory, study population, SmC interventions, barriers or facilitators to smoking cessation interventions, outcomes, limitations, or recommendations.

### Data synthesis

Two reviewers [SI and AK] worked on data analysis and synthesis. The analysis plan was thoroughly discussed with systematic review experts (RB and PP). We also included critical feedback from another Ph.D. scholar and assistant professor [LL] on each step of the analysis. We used two approaches to make inferences about the extracted data. Smoking cessation interventions were analyzed through WHO-FCTC and SmC facilitators and barriers through manual content analysis.

We listed the FCTC articles 6–22 in a column. In the top row, the numbered coded studies were conscripted. We pooled the extracted interventions by aligning them with each relevant FCTC article and below the coded source of literature. We put a “+” or “-” symbol to simplify the data in a tabular form. Two main tables were developed for the SmC interventions; one reflected extractions from empirical sources while the second was from non-empirical sources.

Smoking cessation facilitators and barriers were copied to a Microsoft™ Word document. All barriers and facilitators were color-coded, similar or concomitant facilitators and barriers pooled into categories. Similarly, related categories were synthesized under broader themes.

### Digressions from protocol

To include additional publications, we considered a second (updated) literature search in June 2021 at which time we included the EBSCO CINAHL Complete database instead of CINAHL and EBSCO Dentistry and Oral Sciences.

### Patients and population involvement

There was no involvement of patients or the public in this study.

## Results

**Fig. 1:**
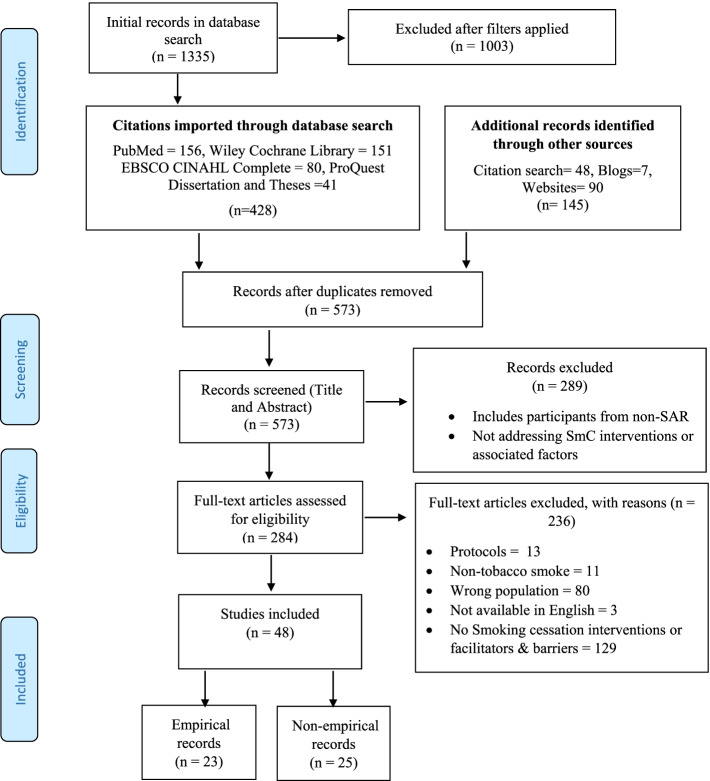
PRISMA Flow Diagram

After de-duplication, the search through databases and grey literature sources yielded 573 citations. As illustrated in Fig. 1, we had 284 citations after reviewing the titles and abstracts of the total citations. With full read, mutual agreement, and discussion with senior faculty members [RB & PP] 48 data sources were included comprised of 23 empirical and 25 non-empirical records.

### Characteristics of included studies

The characteristics of included empirical studies are mentioned and attached as supplementary material-I. Of the 23 empirical records, most (13) were conducted in India, while one study was simultaneously conducted in two countries (Pakistan and Bangladesh). Table 2 provides the countries from which the data sources were traced.

**Table 2:** Origin of included records

Country	India	Pakis-tan	Nepal	Sri Lanka	Bang-ladesh	Bhutan	Maldi-ves	Afghan-istan
No. of empirical records extracted	13	05	04	01	01	00	00	00
	56.56%	21.73%	17.39%	4.34%	4.34%	–	–	–
No. of non-empirical records extracted	05	05	05	02	02	02	02	02
	20%	20%	20%	8%	8%	8%	8%	8%

### Smoking cessation interventions

We analyzed the interventions extracted from both the empirical and non-empirical records through FCTC articles. Of the 22 FCTC strategies (which are called articles), the initial five articles are introductory to the framework while article seven pertains to articles eight to thirteen. Hence, we aligned the extracted interventions with the remaining 16 FCTC articles.

The focus of 73.9% of the empirical studies conducted in SAR in the past 5 years has been on education, communication, training, and public awareness (FCTC article 12). Similarly, 65.2% of the studies are aligned with the focus of article 14 which is demand reduction that includes clinical strategies to facilitate SmC, reduce smoking dependence, and control smoking at institutional levels. Moreover, 52% of the empirical records have been on the protection of tobacco smoke exposure including awareness of smoking health hazards as well as policies and regulations related to smoking-free spaces. Conversely, no empirical records focused on measures related to tobacco price and taxation, regulation of tobacco products, packaging and labeling, illegal trade of tobacco products, cigarettes sale to and by underage, economic alternatives for tobacco workers, protection of environment and health, and reporting and exchange of information. The details pertinent to SmC interventions extracted from empirical records and aligned with FCTC articles appear in Table 3.

**Table 3:** FCTC articles addressed in empirical literature from the South Asian Region

FCTC Articles	Study Codes																							
	QN* 1	QN 2	QN 3	QN 4	QN 5	QN 6	QN 7	QN 8	QN9	QN10	QN 11	QN 12	QN 13	QN14	QN 15	QN 16	QN17	QN 18	QN 19	QL* 1	MM* 1	MM 2	MM 3	Total
6: **Price & tax measures**	–	–	–	–	–	–	–	–	–	–	–	–	–	–	–	–	–	–	–	–	–	–	–	00 (0%)
8: **Tobacco smoke protection**	+	+	–	–	–	+	–	+	–	–	+	+	+	–	+	–	–	–	+	+	–	+	+	**12 (52.17%)**
9: **Regulation of tobacco products content**	–	–	–	–	–	–	–	–	–	–	–	–	–	–	–	–	–	–	–	–	–	–	–	**00 (0%)**
10: **Regulation of tobacco products disclosures**	–	–	–	–	–	–	–	–	–	–	–	–	–	–	–	–	–	–	–	–	–	+	–	**01 (4.34%)**
11: **Packaging & labeling of tobacco products**	–	–	–	–	–	–	–	–	–	–	–	–	–	–	–	–	–	–	–	–	–	–	–	**00 (0%)**
12: **Education, communication, training, and public awareness**	+	+	+	+	+	+	–	+	–	–	+	+	+	–	+	+	–	+	–	+	+	+	+	**17 (73.91%)**
13: **Tobacco advertising, promotion & sponsorship**	–	–	–	–	–	–	–	–	–	–	–	–	–	–	–	–	–	–	–	–	–	–	–	**00 (0%)**
14: **Demand reduction**	+	+	+	+	+	+	–	+	–	–	+	+	+	–	+	+	–	+	+	–	–	–	+	**15 (65.21%)**
15: **Illicit trade in tobacco products**	–	–	–	–	–	–	–	–	–	–	–	–	–	–	–	–	–	–	–	–	–	–	–	**00 (0%)**
16: **Sales to & by minors**	–	–	–	–	–	–	–	–	–	–	–	–	–	–	–	–	–	–	–	–	–	–	–	**00 (0%)**
17: **Economically viable alternative activities**	–	–	–	–	–	–	–	–	–	–	–	–	–	–	–	–	–	–	–	–	–	–	–	**00 (0%)**
18: **Protection of the environment & health**	–	–	–	–	–	–	–	–	–	–	–	–	–	–	–	–	–	–	–	–	–	–	–	**00 (0%)**
19: **Liability**	+	+	+	+	+	+	–	+	–	–	–	–	–	–	–	–	–	+	–	–	+	+	+	11 (47.82%)
20: **Research, surveillance & exchange of information**	–	–	–	–	–	–	–	–	–	–	–	–	–	–	–	–	–	+	–	–	–	–	+	**02 (8.69%)**
21: **Reporting & exchange of information**	–	–	–	–	–	–	–	–	–	–	–	–	–	–	–	–	–	–	–	–	–	–	–	**00 (0%)**
22: **Cooperation in scientific, technical, & legal fields & providing related expertise**	+	–	–	–	–	–	–	+	–	–	+	–	–	–	–	–	–	+	–	–	–	–	+	05 (21.73%)

*QN ± Quantitative, QL* ± Qualitative, MM* ± Mixed Methods Research

Amongst the 25 non-empirical records, 80% addressed tobacco smoke protection that comprises awareness of smoking health hazards and regulations of policies related to smoke-free spaces (article 8). Similarly, 80% of the records mentioned FCTC article 13 which highlights the importance of working on controlling promotion, advertisement, and sponsorship of tobacco. Moreover, tobacco product packaging and labeling (FCTC article 11) is mentioned in 72% of the records. No non-empirical records discussed regulation of contents in tobacco products (article 9), economically sufficient alternatives for tobacco workers (article 17), or research, surveillance, and information exchange (article 20). The detailed record of the interventions extracted from non-empirical sources is presented in Table 4.

**Table 4:** FCTC Articles addressed in Non-Empirical literature from the South Asian Region

FCTC Articles	Data Source Codes																									
	WR^a^ 1	WR 2	WR 3	WR 4	WR 5	WR 6	WR 7	WR 8	WR 9	WR 10	WR 11	WR 12	WR 13	WR 14	WR 15	WR 16	WR 17	WR 18	WR 19	WR 20	WR 21	WR22	WR23	WR24	WR25	Total
6: **Price & tax measures**	+	–	–	–	–	+	+	+	+	+	+	+	+	+	+	+	+	+	+	–	+	–	+	–	–	17 (68%)
8: **Tobacco smoke protection**	+	+	+	–	–	+	+	+	+	+	+	+	+	+	+	+	+	+	+	+	–	–	+	+	+	**20** (80%)
9: **Regulation of tobacco products content**	–	–	–	–	–	–	–	–	–	–	–	–	–	–	–	–	–	–	–	–	–	–	–	–	–	**00 (0%)**
10: **Regulation of tobacco products disclosures**	–	–	–	–	–	–	–	–	–	–	–	–	–	–	–	–	+	–	–	–	+	–	–	–	–	**02 (8%)**
11: **Packaging & labeling of tobacco products**	+	–	–	–	–	+	–	+	+	+	+	+	+	+	+	–	+	+	–	+	+	+	+	+	+	18 (72%)
12: **Education, communication, training, and public awareness**	+	+	–	+	–	–	–	–	+	+	+	+	+	–	–	–	–	–	+	–	–	–	–	–	–	09 (36%)
13: **Tobacco advertising, promotion & sponsorship**	+	–	–	–	–	+	+	+	+	+	+	+	+	+	+	+	+	+	+	+	+	+	+	+	–	**20 (80%)**
14: **Demand reduction**	+	+	–	–	–	–	–	–	–	–	–	–	–	–	–	–	–	–	–	–	–	–	–	–	–	**02 (8%)**
15: **Illicit trade in tobacco products**	+	–	–	–	–	–	–	+	–	–	–	–	–	–	–	–	+	–	–	–	–	–	–	–	–	**03 (12%)**
16: **Sales to & by minors**	–	–	+	–	–	–	–	–	–	–	–	–	–	–	–	–	–	+	+	+	+	–	+	–	–	06 (24%)
17: **Economically viable alternative activities**	–	–	–	–	–	–	–	–	–	–	–	–	–	–	–	–	–	–	–	–	–	–	–	–	–	**00 (0%)**
18: **Protection of the environment & health**	+	–	–	–	–	–	–	–	–	–	–	–	–	–	–	+	–	–	–	–	–	–	–	–	–	**02 (8%)**
19: **Liability**	+	–	–	–	–	–	+	–	–	–	–	–	–	–	+	+	–	–	–	–	–	–	–	–	–	**04 (16%)**
20: **Research, surveillance & exchange of information**	–	–	–	–	–	–	–	–	–	–	–	–	–	–	–	–	–	–	–	–	–	–	–	–	–	**00 (0%)**
21: **Reporting & exchange of information**	+	–	–	–	–	–	–	–	–	–	–	–	–	–	–	–	–	–	–	–	–	–	–	–	–	**01 (4%)**
22: **Cooperation in scientific, technical, & legal fields & providing related expertise**	–	+	–	–	–	–	–	–	–	–	–	–	–	–	–	–	–	–	–	–	–	–	–	–	–	**01 (4%)**

^a^*WR* Website Review

### The focus of empirical and non-empirical literature

Tables 3 and 4 illustrated above show that some of the FCTC articles are taken into consideration by official anti-smoking organizations in SAR, while empirical inquiries taking place in universities and healthcare organizations have been focused on selective articles/strategies of the FCTC. Such discrepancy is summarized in Table 5.

**Table 5:** Discrepancies in the focus of empirical and non-empirical records in SAR

FCTC Articles	Number of Empirical records addressing FCTC articles	Number of Non-empirical records addressing FCTC articles
6: **Price & tax measures**	00 (0%)	17 (68%)
11: **Packaging & labeling of tobacco products**	00 (0%)	18 (72%)
13: **Tobacco advertising, promotion & sponsorship**	00 (0%)	20 (80%)
14: **Demand reduction**	15 (65.21%)	02 (8%)
16: **Sales to & by minors**	00 (0%)	06 (24%)
19: **Liability**	11 (47.82%)	04 (16%)
22: **Cooperation in scientific, technical, & legal fields & providing related expertise**	05 (21.73%)	01 (4%)

The analysis of data has also identified several articles of FCTC that are not focused or minimally focused on by anti-smoking organizations nor by researchers. The almost neglected articles of FCTC in SAR are presented in Table 6.

**Table 6:** FCTC articles not or nominally addressed in empirical and non-empirical records in SAR

FCTC Articles	Number of Empirical records addressing FCTC articles	Number of Non-empirical records addressing FCTC articles
9: **Regulation of tobacco products content**	00 (0%)	00 (0%)
10: **Regulation of tobacco products disclosures**	01 (4.34%)	02 (8%)
15: **Illicit trade in tobacco products**	00 (0%)	03 (12%)
17: **Economically viable alternative activities**	00 (0%)	00 (0%)
18: **Protection of the environment & the health**	00 (0%)	02 (8%)
20: **Research, surveillance & exchange of information**	02 (8.69%)	00 (0%)
21: **Reporting & exchange of information**	00 (0%)	01 (4%)

Finally, the FCTC article uniformly focused on empirical as well non-empirical data sources was to be article 8 i.e. tobacco smoke protection.

### Factors associated with smoking cessation interventions

The 48 included records were reviewed and factors associated with SmC interventions were manually identified through content analysis. Details of codes with frequencies, categories, and themes are provided in supplementary material-II. Factors associated with SmC interventions were divided into barriers and facilitators as discussed herein.

#### Barriers associated with smoking cessation

Barriers were condensed into four themes (Table 7). At the individual level, the most prevalent SmC barrier found in literature from SAR is lack of awareness regarding the harms associated with cigarette smoking. Furthermore, lack of primary resources to facilitate SmC, lack of interest and motivation, conditioning of smoking with certain situations, and hesitation in seeking support to quit are important barriers to SmC interventions in SAR. At the policy level, ineffective implementation of anti-smoking policy and loopholes in these policies reduce the effectiveness of anti-smoking initiatives in SAR. In some countries, there is no restriction on the sale of single-stick cigarettes [29, 30]. Meanwhile, the measures and tactics used by the tobacco industry are also accelerating tobacco use. Despite bans on cigarette smoking in public spaces, there are still widespread exceptions to this guideline as people smoke in non-air-conditioned coffee shops, restaurants, hotels, airports, and many other public spaces in SAR. At the healthcare level, poor accessibility, lack of resources, role ambiguities as well as lack of interest among healthcare professionals (HCPs) regarding SmC are also commonly observed barriers in SAR. Moreover, social acceptance, motivation, or pressure acquired from social gatherings or parental smoking have also been commonly observed barriers in SAR.

**Table 7:** Barriers associated with smoking cessation

At Individual Level	At Institutional Level	At Healthcare Level	At Socio-cultural Level
• Unawareness about smoking harms & SmC strategies [31–37]	• Aberration of anti-smoking laws [29, 30, 36–44]	• Lack of resources for SmC [31, 34, 45, 46]	• Social engagement [31, 46, 47]
• Psychological factors [31, 34, 47–50]	• Loopholes in anti-smoking regulations [38]	• HCPs’ lack of interest in SmC initiatives [38, 51]	• Social acceptability [34, 45, 49]
• Nature of smoking [38, 47]	• Tactics by the tobacco industry [37, 52, 53]	• Role ambiguities [51]	• Smoking as an acquired behavior [31, 36, 38, 46, 47]
• Presence of smoking triggering factors [31, 34, 38, 47, 49, 54–56]			
• Barriers to seeking support for SmC [31, 34, 45, 46, 50]			

#### Facilitators associated with smoking cessation

The SmC facilitators were synthesized in four themes (see Table 8). At an individual level, awareness of smoking-associated harms is found as the most prevalent facilitator to SmC interventions and this is further enhanced with the occurrence of any of the smoking-related health risks like a respiratory or cardiovascular issue. In addition, the role of mass media in increasing awareness regarding tobacco hazards is also acknowledged in literature from SAR. In addition, the guilt of harming others through second-hand smoke and the realization of being a source of imitation for non-smokers also increases the smokers’ intention for cessation. Furthermore, readiness for SmC and planning for coping with withdrawal symptoms are also important facilitators for SmC. At the policy level, effective implementation of policies especially related to increased taxation, smoke-free spaces, health warnings, and graphics are important for reducing the prevalence of cigarette smoking in SAR. In Bhutan, the sale of tobacco products is prohibited in the market [42]; however, in Pakistan, due to poor control of the prevalence of cigarette smoking, there are strict recommendations for implementation of MPOWER strategy and strict implementation of anti-smoking laws [37]. At the healthcare level, the establishment of an anti-smoking facilitation center, availability of SmC trained HCPs, and provision of anti-smoking services in outreached communities are important facilitators of SmC interventions in SAR. In Sri Lanka, community-level SmC strategies are complementary to clinical-level supports for reducing the current prevalence of cigarette smoking [34]. At the sociocultural level in SAR, availability of support systems, like family and friends, discussion related to smoking harms in communities, and consideration of sociocultural values while devising anti-smoking policies are imperative to control smoking prevalence.

**Table 8:** Facilitators associated with smoking cessation

At Individual Level	At Institutional Level	At Healthcare Level	At Socio-cultural Level
• Awareness of smoking-associated harms [31, 49, 50, 57]	• Implementation of anti-smoking laws, rules & regulations [31, 37, 41, 42, 44, 45, 53, 54, 57–59]	• SmC facilitation centers [34, 60]	• Support system [38, 50, 54, 56, 61]
• Occurrence of smoking-related health risks [38, 49, 50, 60, 62]		• HCPs’ training on SmC [31, 33, 38, 51, 54, 62, 63]	• Snowballing reluctance to cigarette smoking in community [31]
• Psychological factors [31, 33, 46, 50, 57, 60, 61]		• HCPs working beyond hospitals [34, 51, 52]	• Socio-cultural considerations [31, 44, 45, 47, 48, 54, 63]

## Discussion

The analysis of data from SAR highlighted important interventions for controlling and/or reducing cigarette smoking. However, the findings suggest that the implementation of FCTC strategies/articles in a uniform way is missing in SAR. There are discrepancies between the research inquiries taking place in universities, hospitals, or other such institutions and the measures taken by different official anti-smoking organizations in SAR. Such discordant directions contribute to a lack of coordination between education, clinical, and anti-smoking organizations’ efforts for smoking cessation in SAR. The need for coordination between clinical and public policy levels has been highlighted in previous literature from south Asian countries [64, 65]. Similarly, a secondary analysis of existing data has shown different countries in South Asia as focusing more on the implementation of specific anti-tobacco policies while lagging in the implementation of others [66].

On the other hand, some of the FCTC articles (refer to Table 6) have been minimally are completely missed in the anti-tobacco efforts in SAR. This delineates the lack of following the Sustainable Development Goal 3A (SDG-3A): which reinforces the implementation of FCTC articles globally [65]. As South Asian countries are the second-highest tobacco products suppliers in the world [66], a priority focus on articles 15 and 17 is immensely important in SAR. However, this review found that these two FCTC articles are not addressed in any of the included empirical studies in this review.

The association between FCTC articles’ implementation and reduced prevalence of tobacco consumption is already reported in literature [66]. However, the current review found only two FCTC articles (8 & 12) addressed in both empirical and non-empirical records at a satisfactory level. Hence, the current review identifies a limited range of anti-smoking initiatives in SAR which is the reason for the majority (84%) of the world’s smokers residing in developing countries and is predicted to grow to 88% in 2025 [66].

Most barriers and facilitators identified in this review aligned with different studies conducted in South and Southeast Asian countries [67–69]. However, HCPs’ lack of interest in anti-smoking activities and confusion regarding their roles and responsibilities in SmC surfaced in the current review. Similarly, gradual increase in reluctance to cigarette smoking and second-hand smoke in community people, and availability of any support system for SmC are imperative factors for reducing smoking prevalence in SAR are also unique findings of the current review. Moreover, the scoping review reveals that despite the realization by governmental and non-governmental organizations of the effectiveness of well-implemented anti-smoking laws, the non-committal attitudes of individuals and some officials compromise the successful implementation of the laws.

The current study signifies the need for strong national-level coordination among anti-tobacco organizations, policymakers, researchers, and educators. Also, there is a need for an updated surveillance system for tobacco prevalence, and tobacco cessation and reduction rates. Moreover, the study highlights the need for mobilization of existing resources for the establishment of smoking cessation cells/departments in different localities. Being healthcare professionals, we consider the initiation of such efforts from the tertiary care hospitals’ level. Such anti-smoking cells/departments must not focused only on assisting smokers in quitting, they should also provide training to healthcare professionals for counseling and facilitating smokers in smoking cessation. Lastly, being signatories to FCTC, the south Asian countries must analyze their performance regarding effective or ineffective implementation of the articles presented by the FCTC.

## Conclusion

The growing prevalence of cigarette smoking in developing countries has been accentuated as it causes multiple physical and economic harms. The need for context-based interventions with consideration of local barriers and facilitators is made apparent. Implementation of FCTC articles and continuous monitoring can certainly address the situation. The current review provides a significant contribution to the extant SmC efforts made in SAR.

## Supplementary Information

**Additional file 1: Supplementary Material-I.** Characteristics of retrieved studies. **Supplementary Material II.** Factors associated with smoking cessation interventions. **Supplementary Material-III.** PubMed. CINAHL. Wiley Cochrane. ProQuest Thesis.

## Data Availability

All data generated or analysed during this study are included in this published article (and its supplementary information files) and is available publically.

## Abbreviations

BMJ-Open: British Medical Journal Open
CADHT: Canadian Agency for Drugs and Technologies in Health
FCTC: Framework Convention on Tobacco Control
HCPs: Healthcare professionals
JBI: Joanna Briggs Institute
LMICs: Low- and middle-income countries
MPOWER: M: monitor tobacco use and prevention policies; P: protect people from tobacco smoke; O: offer help to quit tobacco smoking; W: warn about the dangers of tobacco; E: enforce bans on tobacco advertising, promotion and sponsorship; and R: raise taxes on tobacco
PRISMA: Preferred Reporting Items for Systematic Reviews and Meta-Analyses
SAR: South Asian Region
SmC: Smoking cessation
SDG-3A: Sustainable Development Goal 3A
USA: United States of America
WHO: World Health Organization
