# Supplementary material for: Smoking cessation interventions in South Asian Region: a systematic scoping review
Source: BMC Public Health. 2022 Jun 1;22:1096. doi: 10.1186/s12889-022-13443-y (PMC9158221; doi:10.1186/s12889-022-13443-y)

| **Supplementary Material-I**: Characteristics of retrieved studies | | | | | |
| --- | --- | --- | --- | --- | --- |
| **Study reference-and country** | **Design** | **Population** | **Smoking Cessation interventions** | **Smoking Cessation Barriers** | **Smoking Cessation Facilitators** |
| 1. Bhaumik et al., 2019   [India] | Exploratory qualitative | 21 Acute  coronary syndrome (ACS, or heart attack) patients & care providers | Smoking cessation counseling from community health workers and primary care providers | - Little interest or awareness - Resources’ limitations - Influence of Friends & coworkers - Masculine activity - Embarrassment stops smokers to seek facilitation - Stress relief & enhance decision-making ability, and increase alertness - Source of pleasure | - Family members involvement - High perceived self-efficacy - understanding of tobacco harms - Low level of tobacco acceptance in the community - ban of tobacco use in public - Trained HCPs on tobacco-related health risks & - Temporary quit during illness |
| 2-Elsey et al., 2015  [Pakistan] | Clustered randomized control trial | 1573 patients aged 18 years or older with  suspected pulmonary TB & regular smokers | Behavioral support sessions (WHO-5As plus 30 min structured consultation)  Bupropion therapy plus behavioral support  self-help leaflet on smoking cessation | - Old age Smokers - Heavy smokers - Close to smokers who smoke at work | - TB diagnoses - Health risk suspicion as “teachable moment”. |
| 3-Gamlath et al., 2020  [Sri Lanka] | Quantitative before-and-after study design | 1019 community adult males and females | More than 10 Education sessions by HCW i.e. public health midwives with community agents. Selected at least 20 opinion leaders to disseminate the sessions in their surrounding population | Nil | - Strong community links through local Change agents or opinion leaders - Role of primary healthcare workers |
| 4- Kanakia et al., 2016  [India] | Quantitative cross-sectional study | 424 TB patients | No intervention provided | Nil | - Measures were taken at health care facilities |
| 5-Sundararajan et al., 2020  [India] | Prospective randomized interventional study | 154 adult MI patients | 30 minutes counseling session on discharge  The patient information leaflets | Nil | Nil |
| 6- Shuja et al., 2016  [India] | An open-labeled randomized controlled study | 202 COPD patients | Patient Information Leaflet (PIL) emphasizes on "need for smoking cessation" | - Low level of education | motivating and counseling by health care professionals |
| 7- Elsey et al., 2015  [Nepal] | Mixed-methods research | 151 | behavioral support (BS) intervention for six months | - Health workers do not yet accept cessation as a core part of their job | - Health worker skills and relationships to ensure patient-centered communication for behavior change. - Interventions tailored to the local context - Participatory approaches built on political will |
| 8-Kumar et al., 2017  [India] | Clinical trial | 160 Patients | Physician’s advice to patient and family  Brochure Counselling | Nil | - Physician involvement - TB diagnosis - Social workers or counselors as personnel in the TB management team |
| 9-Nichter et al., 2015  [India] | Mixed- methods study | 280 smokers and their wives | Educational  presentations to community leaders  Print materials to smokers (posters, calendars, pamphlets, stickers)  Smoke-free homes video | - Men’s lack of interest in participation | - Collective efforts of community leaders, women, healthcare |
| 10-Sah et al., 2016  [Nepal] | Cross-sectional study | 205 persons | Nil | - Male gender - Illiteracy - Peer pressure, - For recreation, - Habit - Love alone - Leisure time, - Family problems, - Workload, and stress. - Parental smoking or Adult smoker in the family - Tobacco use as part of their culture | - Knowledge about the harm of tobacco use |
| 11-Sharma et al., 2018  [India] | An open-label randomized controlled trial | 800 TB patients | - NRT (only for six wks) along with behavior change counseling - Counseling alone (harmful effects of smoking on health, economy, and on other family members) | Nil | - Combination therapy had - Higher compliance |
| 12-Prasad, J.B. 2018  [India] | Quantitative descriptive | SR | Nil | - Easy availability | - Considering socio-cultural patterns - Comprehensive national tobacco policy regarding all the stakeholders in public health - Assistance for smoking   cessation integrated into the health care services   - People access to correct information r/t effects of tobacco consumption |
| 13- Raspanti, G. A, 2016  [Nepal] | Quantitative Descriptive method | 1212 | - Nil | - Considering social & cultural perceptions - wide-spread acceptance of tobacco use - Limited resources | - Cultural competent inclusive health messaging - Educational outreach programs - Successful legislative actions |
| 14- Isrctn (2019)  [Bangladesh & Pakistan] | Double-blind randomized parallel-group placebo-controlled interventional trial | 2,388 pulmonary TB patients | Cytisine + Behavioural Support  Placebo + Behavioural Support | - Having friends, family members, or co-workers who smoke | - Strong motivation to quit |
| 15-Narvenkar,(2019)  [India] | An open-label randomized controlled trial | 162 patients | Continued smoking cessation advice | - Nil | - Nil |
| 16-Hejjaji et al (2020)  [India] | Cluster randomized controlled trial | 234 smokers | CHW-led home visits focused on counseling  Text messages  brief education | - Nil | - Nil |
| 17- Razzaq, et al. (2020)  [Pakistan] | Cross-sectional study | 607 participants | Nil | - Unemployed - No media exposure related to warning | - Nil |
| 18- Goel, et al., (2017)  [India] | Cluster, randomized controlled trial | 685 smokers | “ABC for TB” | - Nil | - Nil |
| 19-Irfan, et al., (2016)  [Pakistan] | Cross-sectional  study | 12 969 participants | Doctors training to develop smoking cessation skills to facilitate smokers  50 smoking cessation outreach clinics were established  Media (press advertisements and radio announcements) and setting up health camps in public areas | - Friends/peer pressure - Anxiety - Tobacco dependence - Stress/mood changes - Male predominance in tobacco use | - Pakistani social norms that prevent women from smoking habits |
| 20-Karmachary, et al., (2018)  [Nepal] | Cross-sectional  study | 1073 participants | Nil | - Lack of smoking assessment in clinics - Alcohol consumption | - Support |
| 21-Navya, et al., (2019)  [India] | Mixed-methods research | 425 | Brief advice’ to quit based on five As and five Rs model  Tobacco Cessation Clinic  Quitline or m-Cessation initiative | - Lack of motivation - Lack of co-ordination mechanisms b/w TB program and tobacco/alcohol abuse treatment services - There are multiple people involved in collecting information in certain places and designated staff in other places. - Reluctance to seek cessation services and even refusal to quit tobacco. | - family support - Will power of the patients - Fear of complications |
| 22-Ramesh Kumar, et al., (2020)  [India] | Cluster randomized effectiveness trial | 517 male patients | Bupropion therapy plus standard counseling  Enhanced counseling | - Nil | - Nil |
| 23-Siddiqi, et al., (2021)  [Pakistan] | Longitudinal survey | 6,014 adult smokers | COVID-19 as a ‘teachable moment’  smoking cessation treatment | - limited access to smoking cessation treatments | - COVID-19 restrictions |

| **Supplementary Material II:** Factors associated with smoking cessation interventions | | | | | | |
| --- | --- | --- | --- | --- | --- | --- |
| **Barriers** | | | | | | |
| **Barriers at Individual Level** | | **Barriers at Institutional Level** | | **Barriers at Healthcare Level** | **Barriers at Socio-cultural Level** | |
| **Unawareness about smoking harms and smoking cessation strategies**   - Lack of awareness (4) - little knowledge of smoking alternatives - Illiterate (2)   **Psychological Factors**   - Negligence - lack of motivation - Lack of interest (2) - Male are more reluctant to quit (2)   **Nature of Smoking**   - Smokers till old age - Heavy smokers   **Smoking Triggering Factors**   - Certain behaviors are conditioned with smoking like feeling stressed, fishing, watching TV, Alcohol consumption, etc.) - Smoking is considered a masculine activity - For pleasure after meal and tea - Myths (stress relief, increase decision-making ability, and increase alertness) - Anxiety - tobacco dependence (2) - stress (love alone, workload, family problems) - Unemployed   **Barriers in seeking support for smoking cessation**   - Not seeking help due to fear of stigmatization of being a smoker - people do not prefer discussion regarding smoking as a threat | | **Aberration of anti-smoking laws**   - Availability of low-priced cigarettes (2) - Single sticks sale (2) - small packets of cigarettes--Bang (3) - Poor implementation of smoking restriction in places of public use - Smoking products displays/posters on smoking sale centers - Internet tobacco sale   **Loopholes in Anti-smoking regulations**   - designated smoking areas (8) - Easy availability (no restrictions on the sale of tobacco products via vending machines or the internet)—(4) - No Uniformity and alignment in cessation services   **Tactics by Tobacco Industry**   - The global market power of the tobacco industry - Tactics/measures of the tobacco industry (2) | | **Lack of resources for smoking cessation**   - Poor accessibility to cessation services (2) - limited resources [esp. time and human (2) - Male HP can assess and facilitate females while female HP are not there   **HCPs’ Lack of interest in smoking cessation initiatives**   - HP Lack of interest in a smoking cessation program - Poor coordination in clinics and smoking cessation cells   **Role Ambiguities**   - Difficulty in identifying the one with the intention to quit - Whose is responsible to work on cessation? [Role confusion] (2) | **Smoking as a factor for Social engagement**   - Smoking as a form of Social engagement (friends & co-workers) - Peer pressure or peer influence (3)   **Social Acceptability**   - family and community norms and acceptance - social & cultural acceptance - part of their culture - cultural acceptability - low acceptability for female   **Smoking as an acquired behavior**   - Living or working with smokers - Parental smoking or Adult smoker in the family - having friends, family members, or co-workers who smoke | |
| **Facilitators** | | | | | |  |
| **Facilitators at Individual Level** | **Facilitators at Policy Level** | | **Facilitators at Healthcare Level** | | **Facilitators at Socio-cultural Level** |  |
| **Awareness of smoking-associated harms**   - Awareness of harms (2)   **Occurrence of smoking-related Health risk**   - Illness (TB diagnose)—(2)   **The guilt of putting others at risk of Second-hand smoke**   - Realization of harms of SHS [to others]   **Psychological factors**   - confident, and motivated to quit (3) - willpower - less nicotine-dependent   fear of complications among patients   - be aware and ready for quit challenges - know the force making you quit - be aware of smoking triggers - Develop Coping Strategies - Rewards on quits | **Implementation of anti-smoking rules and regulations**   - COVID-19 restrictions - Ban on smoking in public places (3) and within 50 meters of any school, university, or educational institution, - Ban on sale of single cigarettes and small packets of cigarettes - Inc. Taxes are effective (4) - Large pictorial or graphic health warning (3) - Compliance with smoke-free laws - public policies - timely evaluation of State-wise policy intervention effects - legislative actions - Anti-tobacco regulations (2)- regulation of tobacco content, prohibition of adv, regulation of trade, - The sale of tobacco products is prohibited in Bhutan - Strict implementation of smoke-free laws e.g.   A comprehensive ban on tobacco advertisement, promotion, and sponsorship;   - Prioritizing implementation of smoke-free policies | | **Smoking cessation facilitation Centers**   - Quit Facilitation points - Physician involvement - HCPs assistance in cessation   **HCPs’ training on smoking cessation**   - Smoking cessation Trained HCPs - Primary healthcare workers - Skills - Recognizing and managing at Teachable moments (per-diagnostics period) - Teaching at health screening sites - Motivating and counseling patients - Relationship & communication (behavior change) with patient - Context-based approaches - Complementary approaches like community cessation and clinical cessation - Remove Reminders of Tobacco use - Participatory approaches - Combination therapy is effective in terms of success and compliance (2) - awareness programs   **Working beyond the Hospitals**   - Reaching out to communities - Community-level initiatives (change agents or leaders involvement) - Social workers or counselors as part of the smoking-related health risks management team - Teamwork (community leaders, women GP, HCP, and political support) - all the stakeholders in public health - Outreach educational programs - Health messaging - Coordination in anti-smoking organizations | | **Support system facilitates cessation**   - Family members support - Letting Loved Ones Know You Are Quitting - Support makes it quite easy   **snowballing reluctance to cigarette smoking**   - Low level of tobacco acceptance in the community   **Socio-cultural considerations**   - socio-cultural patterns - Women can’t smoke due to social norms |  |
|  |  |  |  |  |  |  |

**Complementary Material-III**

**PubMed**


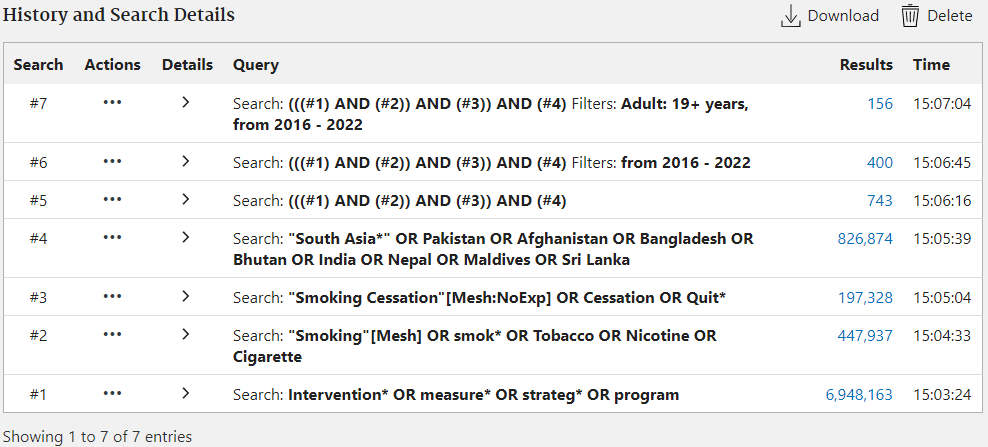


**CINAHL**


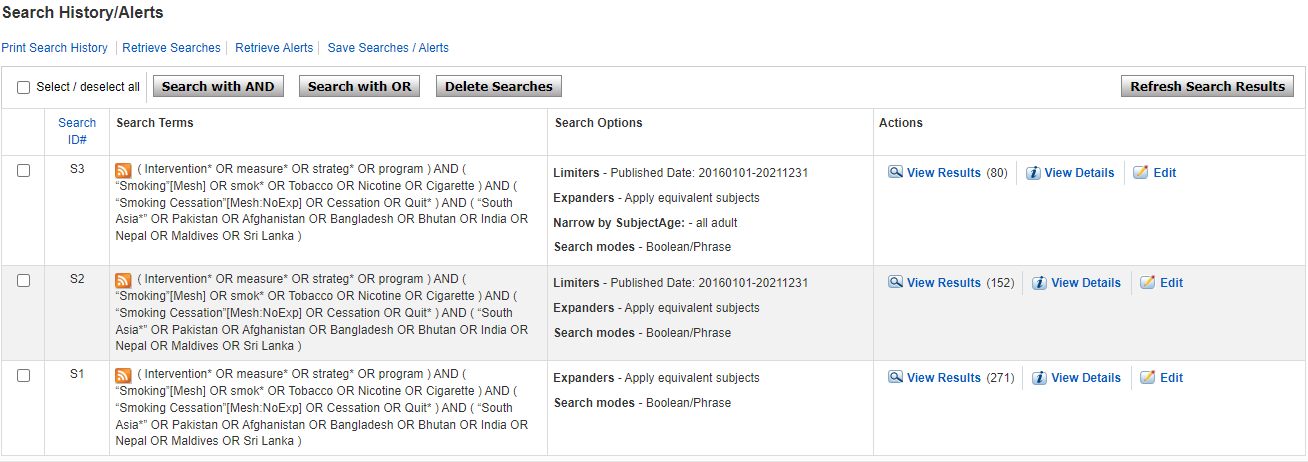


**Wiley Cochrane**


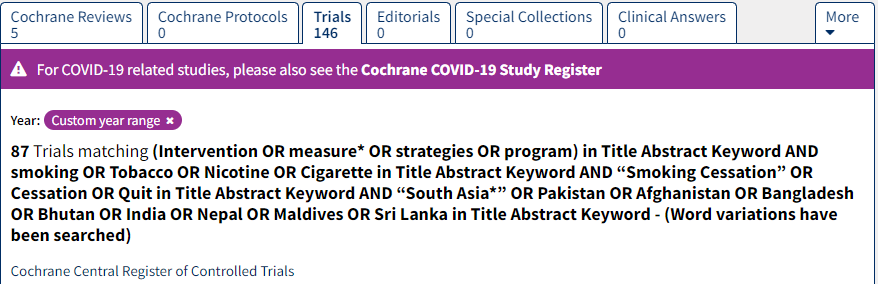


**ProQuest Thesis**


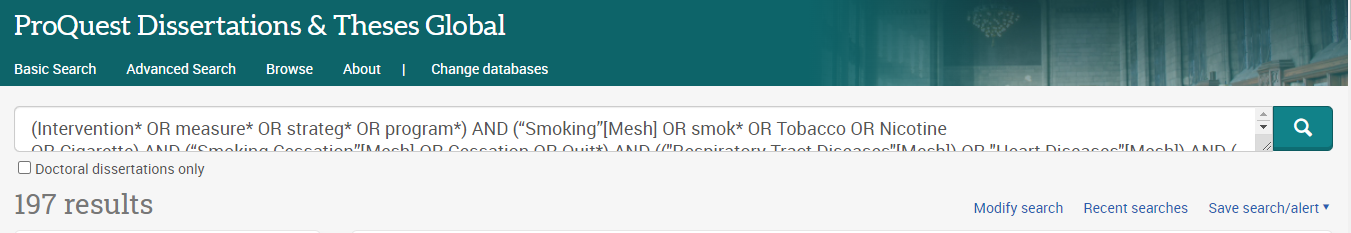


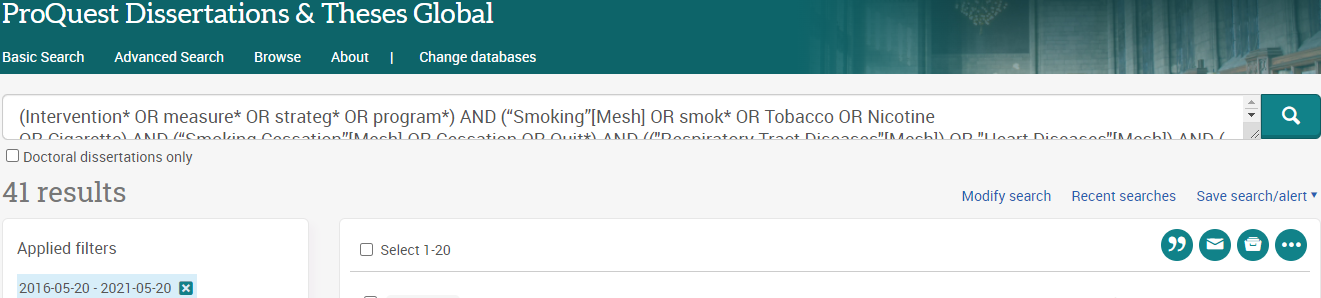

Supplement: Supplementary file 1 — Additional file 1: Supplementary Material-I. Characteristics of retrieved studies. Supplementary Material II. Factors associated with smoking cessation interventions. Supplementary Material-III. PubMed. CINAHL. Wiley Cochrane. ProQuest Thesis. [file 12889_2022_13443_MOESM1_ESM.docx]
